# Evaluation of multilocus marker efficacy for delineating mangrove species of West Coast India

**DOI:** 10.1371/journal.pone.0183245

**Published:** 2017-08-17

**Authors:** Ankush Ashok Saddhe, Rahul Arvind Jamdade, Kundan Kumar

**Affiliations:** 1 Department of Biological Sciences, Birla Institute of Technology & Science Pilani, K. K. Birla Goa Campus, Goa, India; 2 Sharjah Research Academy, University of Sharjah, Sharjah, United Arab Emirates; National Institute of Plant Genome Research, INDIA

## Abstract

The plant DNA barcoding is a complex and requires more than one marker(s) as compared to animal barcoding. Mangroves are diverse estuarine ecosystem prevalent in the tropical and subtropical zone, but anthropogenic activity turned them into the vulnerable ecosystem. There is a need to build a molecular reference library of mangrove plant species based on molecular barcode marker along with morphological characteristics. In this study, we tested the core plant barcode (*rbcL* and *matK*) and four promising complementary barcodes (ITS2, *psbK-psbI*, *rpoC1* and *atpF-atpH*) in 14 mangroves species belonging to 5 families from West Coast India. Data analysis was performed based on barcode gap analysis, intra- and inter-specific genetic distance, Automated Barcode Gap Discovery (ABGD), TaxonDNA (BM, BCM), Poisson Tree Processes (PTP) and General Mixed Yule-coalescent (GMYC). *matK*+ITS2 marker based on GMYC method resolved 57.14% of mangroves species and TaxonDNA, ABGD, and PTP discriminated 42.85% of mangrove species. With a single locus analysis, ITS2 exhibited the higher discriminatory power (87.82%) and combinations of *matK* + ITS2 provided the highest discrimination success (89.74%) rate except for *Avicennia* genus. Further, we explored 3 additional markers (*psbK*-*psbI*, *rpoC1*, and *atpF*-*atpH*) for *Avicennia* genera (*A*. *alba*, *A*. *officinalis* and *A*. *marina*) and *atpF-atpH* locus was able to discriminate three species of *Avicennia* genera. Our analysis underscored the efficacy of *matK* + ITS2 markers along with *atpF-atpH* as the best combination for mangrove identification in West Coast India regions.

## Introduction

Plant DNA barcoding is more complex than animal DNA barcoding and it often requires more than one locus approach. The mitochondrial cytochrome oxidase I (COI) gene fragment is considered as the universal animal barcode. Plant mitochondrial COI was excluded from the barcoding, due to the low substitution rates [1–3]. Later, the Consortium for the Barcode of Life (CBOL) evaluated 7 leading candidate DNA regions (*matK*, *rbcL*, *trnH–psbA* spacer, *atpF–atpH* spacer, *rpoB*, *rpoC1*, and *psbK–psbI* spacer) [4]. The CBOL recommended two-locus combinations of *rbcL* and *matK* as the core plant barcode complemented with *trnH-psbA* intergenic spacer based on the parameters of recoverability, sequence quality, and levels of species discrimination, CBOL [4–6]. China Plant Barcode of Life recommended the internal transcribed spacer (ITS) as an additional candidate plant DNA barcode [7]. Comparative studies of seven markers *psbA-trnH*, *matK*, *rbcL*, *rpoC1*, *ycf5*, ITS2, and ITS from medicinal plant species were performed. Authors recommended that ITS2 is the best potential marker which discriminated 92.7% plants at the species level in more than 6600 plant samples [8]. The potential discriminating DNA barcode varies from one botanical family to other. The plastid marker *matK* can differentiate more than 90% of species in the Orchidaceae (Orchid family) but less than 49% in the Myristicaceae (nutmeg family) [9–10]. However, identification of 92 species from 32 genera using multilocus markers (coding regions (*rpoB*, *rpoC1*, *rbcL*, *matK* and 23S rDNA) and non-coding (*trnH-psbA*, *atpF–atpH*, and *psbK–psbI*) could achieve 69%-71% with several combinations [3]. More than two loci can improve the plant identification success rate; a recent example of the flora of Canada revealed 93% success in species identification with *rbcL* and *matK*, while the addition of the *trnH-psbA* intergenic spacer achieved discrimination up to 95% [11]. *rbcL* and *matK* loci showed poor discrimination in species-rich genera and complex taxa of *Lysimachia*, *Ficus*, *Holcoglossum*, and *Curcuma* [12–15]. The lowest discriminatory power was observed in closely related groups of *Lysimachia* with *rbcL* (26.5–38.1%), followed by *matK* (55.9–60.8%) and combinations of core barcodes (*rbcL* + *matK*) had discrimination of 47.1–60.8% [15]. Beside all these markers, several plastid regions such as *ycf1*, *atpF-H*, *psbK-psbI*, *ropC1*, *rpoB*, and *trnL-trnF* were frequently evaluated as plant barcode. However, the application of DNA barcoding has been hindered owing to the difficulty in distinguishing closely related species, especially in recently diverged taxa.

Mangroves are unique component of the coastal ecosystem of the world with a niche distribution in tropical and subtropical climates [16]. They are adapted to the local environment like fluctuated water level, salinity and anoxic condition through special features such as aerial breathing and extensive supporting roots, buttresses, salt-excreting leaves and viviparous propagules [17–18]. Plant mangrove species comprise 70 species belonging to about 20 families and 27 genera [19–20]. The West Coast of India is more or less steeply shelved, lack major deltas, river estuaries and dominated by sandy and rocky substratum. The West Coast also harbors one of the world’s biodiversity hotspot of Western Ghats in India. It includes the states of Gujarat, Maharashtra, Goa, Karnataka, and Kerala, which harbors 37 species (25 genera under 16 families). The most dominant mangrove species found along the West Coast of India are *Rhizophora mucronata*, *R*. *apiculata*, *Bruguiera gymnorrhiza*, *B*. *parviflora*, *Sonneratia alba*, *S*. *caseolaris*, *Cariops tagal*, *Heretiera littoralis*, *Xylocarpus granatum*, *X*. *molluscensis*, *Avicennia officinalis*, *A*. *marina*, *Excoecaria agallocha* and *Lumnitzera racemosa* [21].

In the previous study, we reported the efficacy of single and concatenation of *rbcL* and *matK* marker which resolved *Acanthus*, *Excoecaria*, *Aegiceras*, *Kandelia*, *Ceriops* and *Bruguiera* genus perfectly, but were unable to delimit species-rich genera such as *Rhizophora*, *Avicennia* and *Sonneratia* [17]. In the present work, we comprehensively evaluated the potential of ITS2, concatenated ITS2+*matK*, *atpF-atpH*, *psbK-psbI* and *ropC1*markers for 14 mangroves species. The evaluation was based on genetic distance, diagnostic nucleotide characters, Neighbour-joining (NJ) Kimura 2 Parameter (K2P) tree, TaxonDNA, Automated Barcode Gap Discovery (ABGD), Poisson tree process (PTP) and Generalized mixed Yule- Coalescent model (GMYC) analysis.

## Material and methods

### Ethics statement

The mangrove samples were collected from different parts of Goa, west coast region, with the permission from the Principal Chief Conservator of Forest, Goa Forest Department, Goa, India. Further, none of the species are endangered or protected species.

### Mangrove plant sampling

In the present study, a total of 44 specimens of mangroves belonging to 14 species, 9 genera and 5 families were collected from Goa region, West Coast of India with geographical co-ordinates latitude of 15.5256° N and longitude of 73.8753° E. The selected genera of mangroves such as *Rhizophora*, *Bruguiera*, *Avicennia*, and *Sonneratia* each represented by at least two species and *Aegiceras*, *Excoecaria*, *Ceriops*, *Kandelia*, *Acanthus* genus were represented by single species. Mangrove species were identified based on morphological keys [22] and mounted on herbarium sheets, photographed and deposited at the Botanical Survey of India, Western Regional Centre, Pune, India as barcode vouchers [17]. The well-identified voucher specimens along with their taxonomic information, collection details, and GenBank accession numbers were described in Table 1. For each specimen, leaf tissue was collected in the field, labeled and stored in -80° C for further analysis.

**Table 1:** Details of the mangrove species.

S. No.	Specimen	Voucher No.	Accession No. of ITS2	
**A**				
**1**	*Avicennia officinalis*	AAS-100-02	KU876892, KU876893	
**2**	*Avicennia marina*	AAS-110-12	KU876889, KU876890, KU876891	
**3**	*Avicennia alba*	AAS-120-22	KU876886, KU876887, KU876888	
**4**	*Acanthus ilicifolius*	AAS-230-32	KY250442, KY250443	
**5**	*Bruguiera cylindrica*	AAS-130-32	KU876894, KU876895, KU876896	
**6**	*Bruguiera gymnorrhiza*	AAS-140-42	KU876897, KU876898, KU876899	
**7**	*Rhizophora mucronata*	AAS-150-52	KU876910, KU876911, KY250446	
**8**	*Rhizophora apiculata*	AAS-160-62	KU876908, KU876909, KY250445	
**9**	*Kandelia candel*	AAS-190-92	KU876906, KU876907, KY250444	
**10**	*Ceriops tagal*	AAS-200-02	KU876900, KU876901, KU876902	
**11**	*Excoecaria agallocha*	AAS-180-82	KU876903, KU876904, KU876905	
**12**	*Aegiceras corniculatum*	AAS-170-73	KU876881, KU876882, KU876883, KU876884	
**13**	*Sonneratia caseolaris*	AAS-220-22	KY250450, KY250451	
**14**	*Sonneratia alba*	AAS-210-12	KY250447, KY250448, KY250449	
**B**				
**S. No.**	Specimen	*atpF-atpH*	*psbK-psbI*	*rpoC1*
**1**	*Avicennia officinalis*	KY754573, KY754574, KY754575	KY754564, KY754565, KY754566	KY754187, KY754188, KY754189
**2**	*Avicennia marina*	KY754570, KY754571, KY754572	KY754561, KY754562, KY754563	KY754184, KY754185, KY754186
**3**	*Avicennia alba*	KY754567, KY754568, KY754569,		

Details of the mangrove species with accession numbers used in the present study for ITS2, *atpF-atpH*, *psbK-psbI* and *rpoC1* with voucher number and GenBank accession numbers.

### DNA extraction

Genomic DNA was isolated from mangrove species by modified cetyl-trimethyl ammonium bromide (CTAB) protocol [17]. Leaf tissue was homogenized in liquid nitrogen and CTAB buffer containing 2% PVP-30 and 1% β-mercaptoethanol was mixed. The suspension was incubated at 60°C for 60 min and centrifuged at 14000 rpm for 10 min at room temperature. It was further extracted with equal volume of chloroform: isoamyl alcohol (24:1) and precipitated with cold isopropanol (-20°C) and ammonium acetate. The precipitated DNA was washed with 70% ethanol and finally dissolved in TE buffer. Quantity and quality of the DNA samples were confirmed by agarose gel electrophoresis and Nanodrop (Thermo Scientific, USA).

### PCR and sequencing

PCR amplification of ITS2, *atpF-atpH*, *psbK-psbI* and *rpoC1* were carried out in the 50-μl reaction mixture containing 10-20ng of template DNA, 200 μM of dNTPs, 0.1 μM of each primer and 1 unit of Taq DNA polymerase (Thermo Scientific, USA). The reaction mixture was amplified in Bio-Rad (T100 model) thermal cycler with temperature profile for ITS2 (94°C for 4 min; 35 cycles of 94°C for 30 sec, 56°C for 30 sec, 72°C for 1 min; final extension 72°C for 10 min), *atpF-atpH* (94°C for 1 min; 35 cycles of 94°C for 30 sec, 50°C for 40 sec, 72°C for 40 sec; final extension 72°C for 5 min), *psbK-psbI* (94°C for 5 min; 35 cycles of 94°C for 30 sec, 55°C for 30 sec, 72°C for 45 sec; final extension 72°C for 10 min), *rpoC1* (94°C for 5 min; 35 cycles of 94°C for 30 sec, 55°C for 30 sec, 72°C for 45 sec; final extension 72°C for 10 min). The amplified products were separated by agarose gel (1.2%) electrophoresis and stained with ethidium bromide. The primers used for amplification were listed (Supporting information S1 Table). PCR products were purified as per manufacturer’s instruction (Chromous Biotech) and further sequencing reactions were carried out using the Big Dye Terminator v3.1 Cycle Sequencing Kit (Applied Biosystems) and analyzed on ABI 3500xL Genetic Analyzer (Applied Biosystems).

### Data analysis

Sequence assembly and alignment were performed in Codon Code Aligner v.3.0.1 (Codon Code Corporation) and MEGA 6.0.6 respectively [23]. All sequences were submitted to Barcode of Life Data Systems (BOLD) database under the project code IMDB with their taxonomic and sampling details (doi:10.5883/DS-IMDBNG) [24]. Nucleotide diagnostic characters of mangrove species were analyzed based on the BOLD system. Further, *matK* and ITS2 sequences were concatenated using DNASP v5.10 tool and analyzed in MEGA 6 [25]. NJ trees were constructed using MEGA 6.0 and Kimura 2 parameter (K2P) genetic distance model with node support based on 1000 bootstrap replicates.

### TaxonDNA

TaxonDNA v1.6.2 analysis for species identification with ‘Best Match’ and ‘Best Closest Match’ method was performed [17, 26]. The threshold (T) was set at 95%. All the results above the threshold (T) were treated as ‘incorrect’. Similarly, if all matches of the query sequence were below threshold (T), the barcode assignment was considered to be the ‘correct’ identification. If the matches of the query sequences were good and corresponded to a mixture of species, then it was treated as ambiguous identification.

### Automated Barcode Gap Discovery (ABGD)

The ABGD, is a web server based distance method, which can partition the sequences into potential species based on the barcode gap whenever the divergence within the same species is smaller than organisms from different species [27–29]. The ABGD analysis was performed with two relative gap width (X = 1.0, 1.5) and three distance metrics (Jukes-Cantor, K2P, and p-distance) with default parameters.

### General Mixed Yule-coalescent (GMYC)

The GMYC method requires a fully resolved ultrametric tree for analysis. This Bayesian tree was built using BEAST v1.8 [30–31]. Input file (XML) for BEAST was compiled in BEAUti v1.83 with an HKY+G molecular evolutionary model for the ITS2 dataset and GTR+G for concatenated dataset of *matK*+ITS2. These models were derived using PartitionFinder V1.1.1. Tree prior was set to Yule Process and the length of Markov chain Monte Carlo (MCMC) chain was 40,000,000 generation and sampling was performed at every 4000 step. However, all other settings were kept as default. Convergence of the BEAST runs to the posterior distribution. The adequacy of sampling (based on the Effective Sample Size (ESS) diagnostic) was assessed with Tracer v1.4. After removing the first 20% of the samples as burn-in, all other runs were combined to generate posterior probabilities of nodes from the sampled trees using TreeAnnotator v1.7.4. Estimation of the number of species included in the tree was analyzed using GMYC with single and multiple thresholds in R by the APE and SPLITS packages [27, 30–36].

### Poisson Tree Process model (PTP)

The PTP model is a tree-based method that differentiates specimen into populations and species level using coalescence theory [27–29] The RaxML tree was constructed using CIPRES portal and input data was generated for bPTP analysis. The calculations were conducted on the bPTP web server (http://species.h-its.org), with the following parameters (500,000 MCMC generations, thinning 100 and burn-in 25%).

## Results

### Sequence analysis

**Fig 1:**
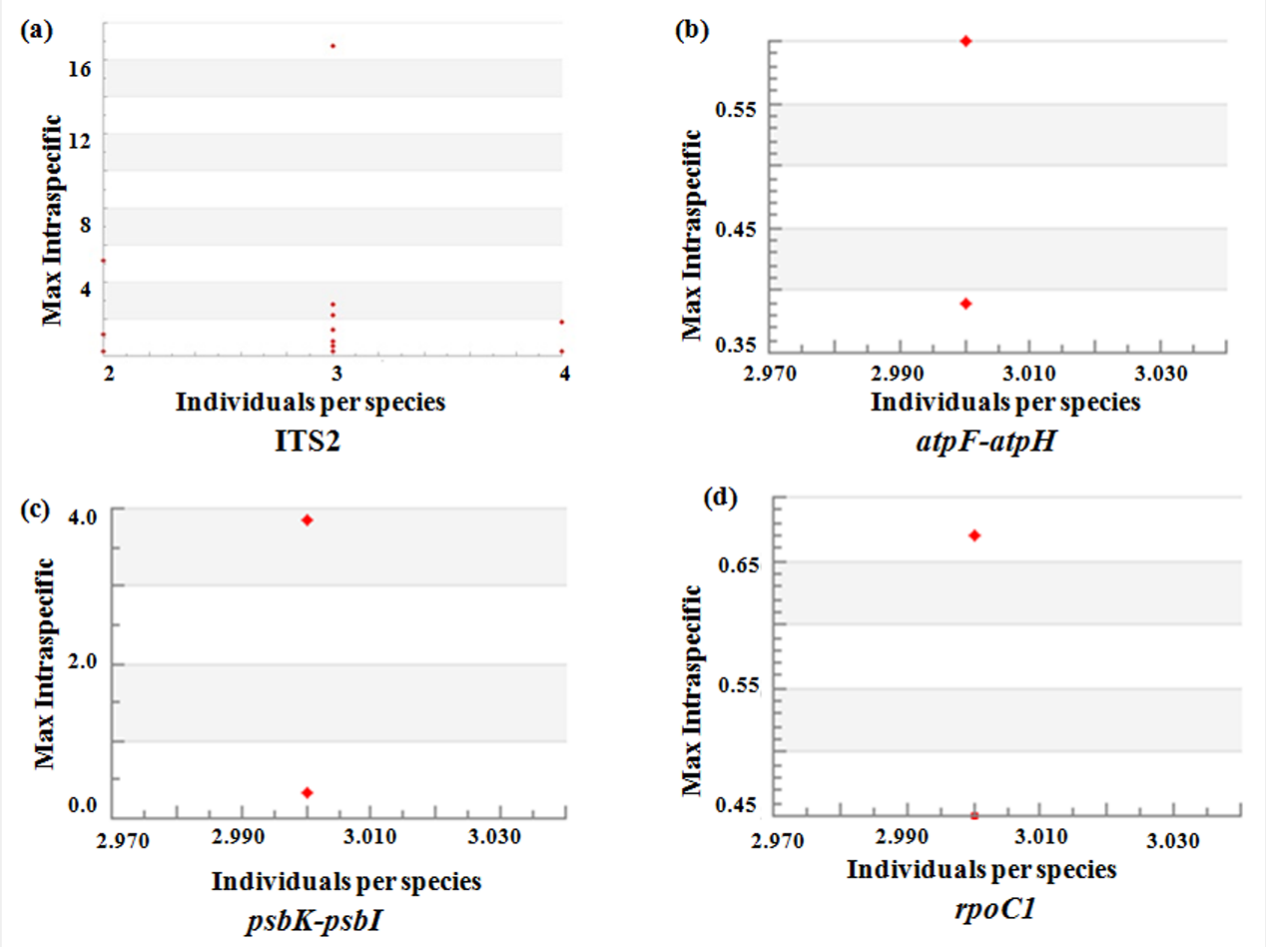
Scatter plot. The scatter plot represents the number of individuals in each species against their maximum intra-specific distances, as a test for sampling bias. (a) ITS2 locus (b) *atpF-atpH* locus (c) *psbK-psbI* locus and (d) *rpoC1* locus.

A total of 148 sequences (44 *rbcL*, 43 *matK*, 40 ITS2, 9 *atpF-atpH*, 6 *psbK-psbI* and 6 *rpoC1*) were acquired from 44 specimens of mangrove belonging to 14 species, 9 genera, and 5 families. The sequences (*rbcL*: 510bp, *matK*: 712bp, ITS2: 445bp, *atpF-atpH*: 511bp, *psbK-psbI*: 360bp and *rpoC1*: 451bp) with few insertions and deletions, without stop codon, along with the specimen collection details were submitted to the Barcode of Life Data Systems (BOLD) in form of a project ‘IMDB’ (dx.doi.org/10.5883/DS-IMDBNG). These sequences were submitted to the NCBI GenBank through BOLD systems and their accession numbers were obtained (Table 1). The scatter plot represented the number of individuals in each species against their maximum intra-specific distances, as a test for sampling bias (Fig 1). Previous evaluation of DNA barcode using *rbcL* and *matK* demonstrated 47.72% and 72.09% efficiency in resolving mangrove taxa respectively. The *matK* sequence region showed better efficiency than the *rbcL* for resolution of mangrove taxa [17]. In the present study, *matK* along with ITS2 and few supplementary markers (*atpF-atpH*, *psbK-psbI* and *rpoC1*) were used for the species identification of the cryptic mangrove taxa. Sequence analysis was performed to estimate the average GC content of the corresponding locus. The average GC content observed was 63.11%, 42.7%, 35.18%, 31.22% and 44.6% for ITS2, *matK*+ITS2, *atpF-atpH*, *psbK-psbI* and *rpoC1* locus respectively.

### Genetic divergence analysis

**Fig 2:**
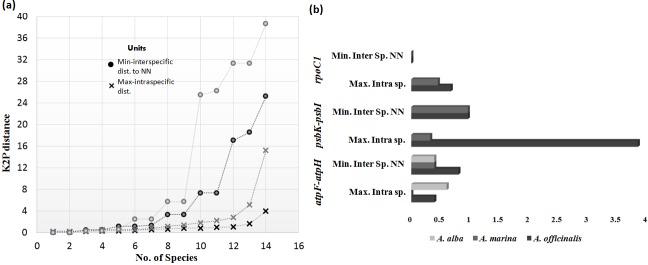
Genetic distance. Distribution of intra and inter specific K2P mean divergence (arranged in ascending order). (a) ITS2 and ITS2+*matK* (concatenated) are represented by grey and black colors respectively. (b) For *atpF-atpH*, *psbK-psbI and rpoC1* markers maximum intraspecific distance and minimum interspecific distance to nearest neighbor are represented by a bar.

The genetic distances were calculated for individual barcode marker by K2P model on the BOLD system. The mean intraspecific distance for ITS2, *atpF-atpH*, *psbK-psbI* and *rpoC1* was calculated as 1.85%, 0.11%, 1.63% and 0.37% respectively. While mean intrageneric distance for ITS2, *atpF-atpH*, *psbK-psbI* and *rpoC1*was calculated as 5.8%, 1.03%, 2.16% and 0.3% respectively (Table 2). Higher intraspecific distances (>2%) for ITS2 were observed in 19.51% individuals and *S*. *alba* exhibited highest intraspecific distance of 16.75%. While lower intrageneric distances (<2%) for ITS2 were observed in 50.98% individuals and *A*. *marina* showed the lowest intrageneric distance of 0%. Higher intraspecific distances for *matK*+ITS2 were observed in 9.30% individuals and *S*. *alba* exhibited the highest distance of 4.01%. While lower intrageneric distances were observed in almost 90.69% individuals (Table 2). In some species intraspecific distance was higher than the intrageneric distance (Fig 2A and 2B). Six species (*A*. *alba*, *A*. *officinalis*, *A*. *marina*, *B*. *cylindrica*, *B*. *gymnorrhiza* and *R*. *mucronata*) were resolved with ITS2, while in concatenation of *matK+*ITS2, error rates were minimized in two species (*A*. *officinalis* and *A*. *marina*). *Avicennia* genus in the former and current analysis has revealed low resolution. To resolve this cryptic genus, we used few supplementary markers such as *atpF-atpH*, *psbK-psbI* and *rpoC1*. *Avicennia* genus showed intraspecific distance ranging from 0%-1.0% with almost all barcode markers, with highest intraspecific distance (>2%) was observed in *psbK*-*psbI* (3.85%) (Fig 2B, Table 3). While lower intrageneric distance (<2%) was observed in nearly all barcode markers, except for *psbK-psbI* (4.94%).

**Table 2:** Distance summary.

Barcode	Level	N	Taxa	Comparisons	Min Dist (%)	Mean Dist (%)	Max Dist (%)	SE Dist (%)
**ITS2**	Species	40	14	39	0	1.85	16.75	0.1
	Genus	25	4	45	0	5.8	35.14	0.25
	Family	28	2	133	5.72	12.35	40.26	0.08
***matK* + ITS2**	Species	39	14	37	0	0.51	4.02	0.02
	Genus	24	4	43	0	1.76	7.84	0.05
	Family	28	2	133	3.35	7.39	19.89	0.03
***atpF-atpH***	Species	9	3	9	0	0.11	0.6	0.02
	Genus	9	1	27	0.39	1.03	1.62	0.02
***psbK-psbI***	Species	6	2	6	0	1.63	3.85	0.27
	Genus	6	1	9	0.96	2.16	4.94	0.14
***rpoC1***	*Species*	6	2	6	0.22	0.37	0.67	0.03
	Genus	6	1	9	0	0.3	0.67	0.02

Summary distribution of sequence divergence at the species, genus and family level is summarized (Distance summary result—BOLD system). N—Number of sequences.

**Table 3:** Mean divergence of *Avicennia* genus.

	*atpF-atpH*		*psbK-psbI*		*rpoC1*	
	Max. Intraspecific	Min Interspecific NN	Max. Intraspecific	Min Interspecific NN	Max. Intraspecific	Min Interspecific NN
***A*. *officinalis***	0.39	0.8	3.85	0.96	0.67	0
***A*. *marina***	0	0.39	0.32	0.96	0.45	0
***A*. *alba***	0.6	0.39	NA	NA	NA	NA

Distribution of intra and inter specific K2P mean divergence for *atpF-atpH*, *psbK-psbI and rpoC1* are represented in table for *Avicennia* genus. NN-Nearest Neighbor, Max-Maximum, Min-Minimum.

Diagnostic character based delineation of mangrove species was done using four barcode markers (ITS2, *atpF-atpH*, *psbK-psbI* and *rpoC1*) along with concatenated *matK*+ITS2 with minimum 3 specimens per species. Highest diagnostic characters were observed in ITS2 for *Excoecaria agallocha* (34) and *Aegiceras corniculatum* (35), whereas single diagnostic character was observed in the species of *Avicennia* genera followed by *Bruguiera cylindrica* (Table 4). In concatenated *matK*+ITS2, highest diagnostic characters were observed in *Aegiceras corniculatum* (96) and *Excoecaria agallocha* (60). However, all species of *Avicennia* genera revealed diagnostic characters, but *Bruguiera gymnorrhiza* failed to exhibit any diagnostic character. The supplementary marker *rpoC1* failed to show any diagnostic character in *Avicennia*, while *atpF-atpH* and *psbK-psbI* exhibited diagnostic characters (Table 4).

**Table 4:** Diagnostic characters of mangrove taxa.

Barcode	Group Name (sequences)	Diagnostic Characters	Diagnostic or Partial Characters	Partial Characters	Partial or Uninformative Characters
***matK*+ ITS2**	*Aegiceras corniculatum* (6)	96	3	0	1
	*Avicennia alba* (3)	8	0	0	1
	*Avicennia marina* (3)	5	0	1	1
	*Bruguiera cylindrica* (3)	2	0	0	0
	*Bruguiera gymnorrhiza* (3)	0	1	0	0
	*Ceriops tagal* (3)	5	2	0	0
	*Excoecaria agallocha* (3)	60	3	0	3
	*Kandelia candel* (3)	12	0	1	1
	*Rhizophora apiculata* (3)	2	0	1	23
	*Rhizophora mucronata* (3)	6	0	0	0
**ITS2**	*Aegiceras corniculatum (4)*	35	4	0	0
	*Avicennia alba (3)*	1	0	1	0
	*Avicennia marina (3)*	1	0	1	0
	*Avicennia officinalis (3)*	0	0	0	0
	*Bruguiera cylindrica (3)*	1	0	0	0
	*Bruguiera gymnorrhiza (3)*	0	0	0	0
	*Ceriops tagal (3)*	4	1	0	0
	*Excoecaria agallocha (3)*	34	2	0	1
	*Kandelia candel (3)*	5	0	1	1
	*Rhizophora apiculata (3)*	2	0	0	1
	*Rhizophora mucronata (3)*	6	1	0	0
***atpF-atpH***	*Avicennia alba (3)*	0	0	0	0
	*Avicennia marina (3)*	4	0	0	0
	*Avicennia officinalis (3)*	2	0	0	0
***psbK-psbI***	*Avicennia marina (3)*	3	0	5	40
	*Avicennia officinalis (3)*	3	0	1	13
***rpoC1***	*Avicennia marina (3)*	0	0	1	0
	*Avicennia officinalis (3)*	0	0	0	0

Identification of diagnostic nucleotides for each of the 14 mangrove taxa recovered from the BOLD system. Based on their utility for mangrove taxa delineating referred as diagnostic characters, diagnostic or partial character, partial characters and partial or uninformative characters.

### Taxonomic assignment

Altogether 40 DNA barcodes from ITS2 and *matK*+ITS2 were used for species delineation. The Neighbour-Joining (K2P) trees constructed with bootstrap support (1000) and bootstrap values of >60 exhibited substantial resolution among the OTUs corresponding to their genera except for *A*. *marina* and *A*. *officinalis* (Supporting information S1 Fig).

### Species identification based on barcoding gap

**Fig 3:**
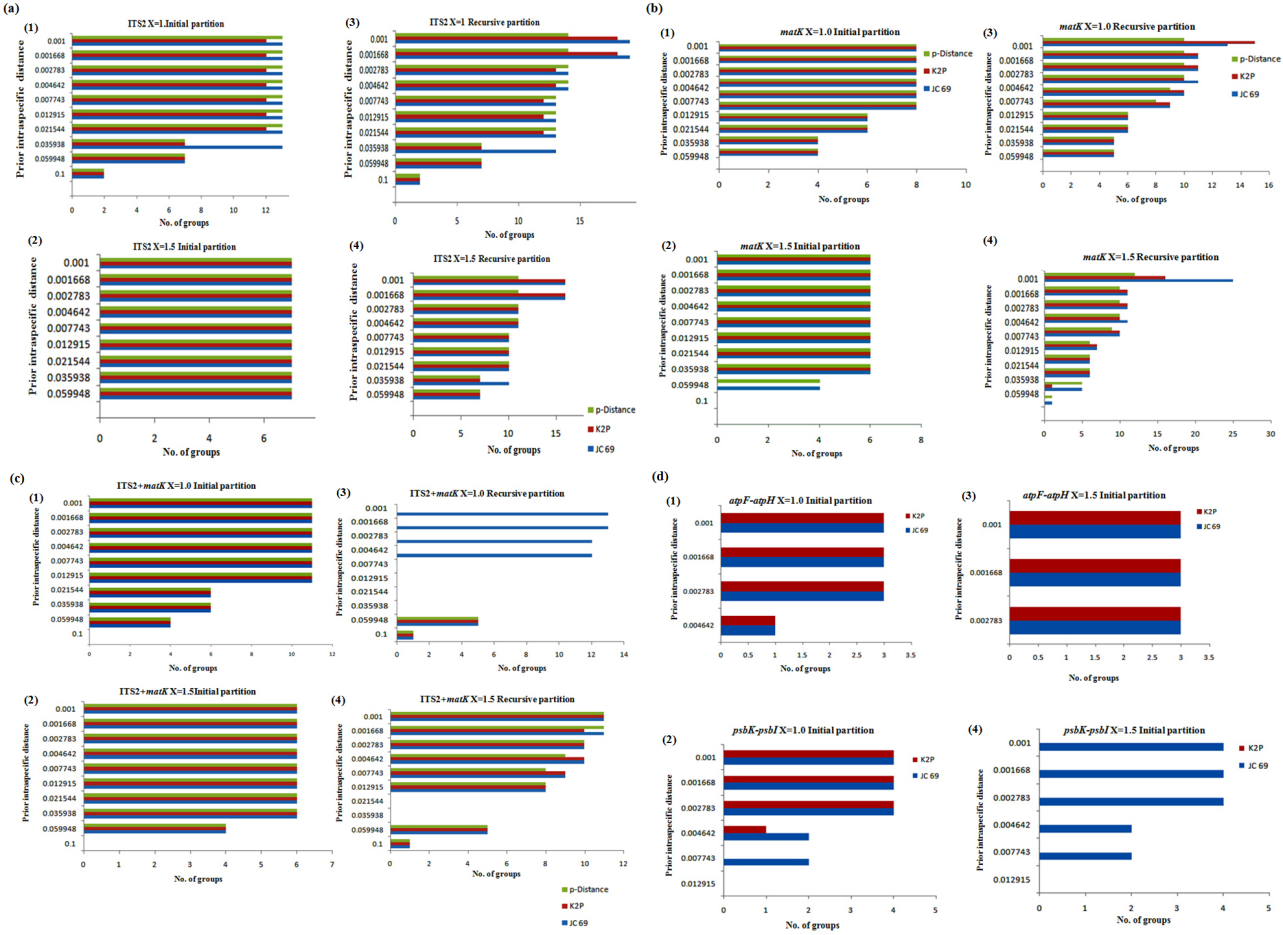
Automated partition. The automatic partition by ABGD with three metrics (JC69, K2P and p-distance) and two X-values (X = 1, 1.5) for (a) ITS2 (initial partition 1,2 and Recursive partition 3 and 4); (b) *matK* (initial partition 1,2 and Recursive partition 3 and 4); (c) ITS2+*matK* (initial partition 1,2 and Recursive partition 3 and 4);(d) *atpF-atpH* and *psbK-psbI* (initial partition 1,2 for *atpF-atpH* and Initial partition 3 and 4 for *psbK-psbI*).

**Fig 4:**
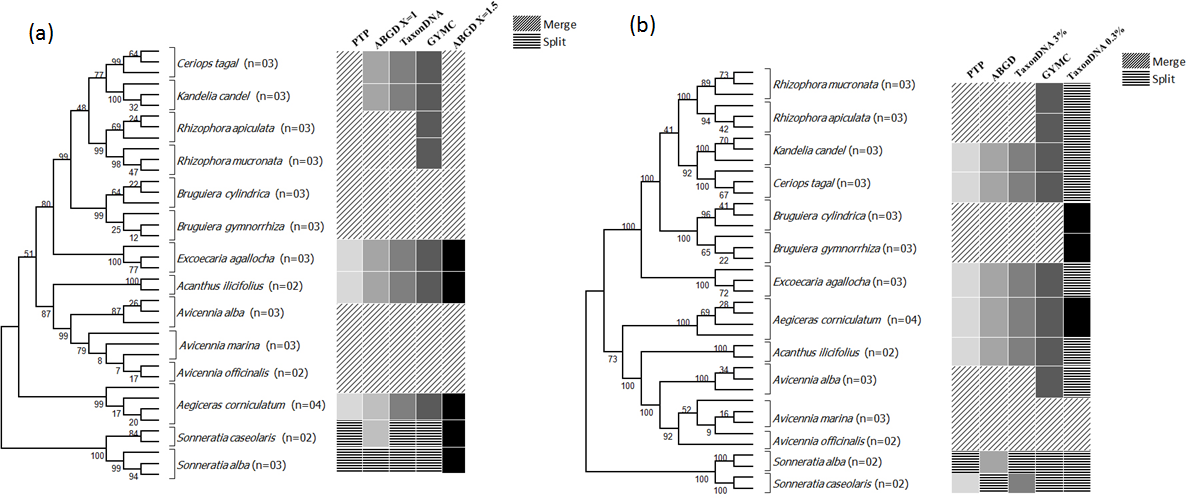
Bayesian phylogenetic tree. Bayesian phylogenetic tree of (a) ITS2 and (b) *matK*+ITS2 gene. Vertical boxes on the right indicate the clades detected by the coalescent-based GMYC, PTP, the distance-based ABGD and TaxonDNA methods.

The initial partition of ITS2, K2P with X = 1.0, prior maximal distance *P* = 0.021 produced consistent 12 operational taxonomic units (OTUs). *S*. *alba* was split into 3 groups, while members of *Rhizophora* and *Avicennia* were merged (Fig 3; Supporting information S2 Table). Whereas, recursive partitioning with *P* = 0.00167, produced inconsistently18 OTUs, of which *A*. *alba*, *A*. *officinalis*, and *B*. *cylindrica* showed split, while *B*. *gymnorrhiza* was clustered perfectly (Fig 4A). In concatenated *matK*+ITS2, at X = 1.0 for all three metrics, OTUs ranged from 4–11 in the initial partition, but recursive partition tends to exhibit inconsistent OTUs (Fig 4B).

When relative gap width was increased from X = 1.0 to X = 1.5, suddenly OTUs in ITS2 for initial partition was dropped to maximum 7, while recursive partition showed an increase in OTUs, up to 16 at *P* = 0.001. The initial partition for *matK*+ITS2, with X = 1, *P* = 0.0129 produced 11 OTUs. *Avicennia* and *Bruguiera* members were merged, while *S*. *alba* showed split. In recursive partition, with *P* = 0.001, *A*. *alba*, *B*. *cylindrica*, *B*. *gymnorrhiza* were resolved perfectly, while *A*. *officinalis*, *A*. *marina* along with *R*. *apiculata and R*. *mucronata* remained merged.

The initial partition with an *atpF-atpH* barcode, JC and K2P metrics with (X = 1, 1.5) showed 3 OTUs (*P* = 0.0027) without any recursive partition except (X = 1.5, *P* = 0.00278, 1 OTU). With *atpF-atpH*, at X = 1.5 initial partition with *P* = 0.00278, 3 OTUs were produced in *A*. *alba*, *A*. *officinalis*, and *A*. *marina*. Similarly, *psbK-psbI* showed 4 OTUs (*P* = 0.001) in an initial partition for JC and K2P metrics at X = 1 and p-distance had only 2 OTUs with 1 OTU in the recursive partition. At X = 1.5, only JC and p-distance were able to partition data. JC the initial partition at *P* = 0.001 produced 4 OTUs, while at *P* = 0.0046, produced 2 OTUs. Metrics p-distance predicted 2 OTUs in an initial partition and 1 OTU in the recursive partition. Barcode locus *rpoC1* at X = 1 with JC and K2P metrics showed initial partition of 2 OTUs and the recursive partition at *P* = 0.00278 predicted 1 OTU.

### Species identification and assignment based on TaxonDNA

The single barcode marker ITS2 produced a moderate rate of correct identification using BM (87.8%) and BCM (75.6%) than the concatenated *matK*+ITS2 using BM (89.74%), and BCM (84.61%) (Table 5). ITS2 barcode produced 13 clusters at 3% threshold, of which 5 species (*A*. *corniculatum*, *A*. *ilicifolius*, *E*. *agallocha*, *K*. *candel* and *C*. *tagal*) were the perfect match. Whereas, *Avicennia*, *Rhizophora* and *Bruguiera* species were clumped into 3 clusters, while *S*. *alba* and *S*. *caseolaris* were split into 5 clusters. As compared to single barcode marker (ITS2), concatenated (*matK*+ITS2) markers at 3% threshold produced 11 clusters, where *S*. *caseolaris* was successfully resolved. Single barcode *atpF-atpH* demonstrated 100% correct identification in both BM and BCM method for *Avicennia* genera with 3 clusters. *psbK-psbI* locus identified 50% *Avicennia* species in BM and BCM methods, however, *rpoC1* showed lowest identification rate of about 33.33% (Table 5).

**Table 5:** TaxonDNA analysis.

Barcodes	No. of Sequences	Best Match (%)			Best Closest match (%)				T (%)	No of Cluster	Match / Mismatch
		C	A	Inc	C	A	Inc	No match			
**ITS2**	40	87.8	2.43	9.75	75.6	2.43	9.75	12.19	3	14	10/4
**ITS2 *+ matK***	39	89.7	2.56	7.6	84.6	2.56	7.6	5.12	3	11	6/8
***atpF-atpH***	9	100	0	0	100	0	0	0	0.3	3	3/0
***psbK-psbI***	6	50	0	50	50	0	50	0	0.8	4	1/1
***rpoC1***	6	33.33	66.66	0	33.33	66.6	0	0	3	1	0/2

TaxonDNA is an alignment-based method based on sequence distance matrices. Percentage of correct/incorrect/ambiguous assignment of a taxon is compared using the molecular operating taxonomic unit (MOTU). The species-specific clustering was performed using match and mismatch criteria. T -Threshold; C–Correct; A–Ambiguous; Inc–Incorrect.

### Species identification and assignment based on GMYC and PTP

The single threshold GMYC (sGMYC) model generated through BEAST using the ultrametric phylogenetic tree resulted in an identification of 9 Maximum Likelihood (ML) clusters for ITS2 with confidence interval (CI) of 4–9 and 14 ML entities with CI of 4–18 (Threshold time: -0.013035). Similarly, with *matK*+ITS2, 10 ML clusters with CI of 4–10 and 14 ML entities with CI of 4–16 (Threshold time: -0.005793) were identified. The resulting ML entities in ITS2 exhibited 5 species merged in 2 OTUs, while in *matK*+ITS2 only 4 species were merged with exception of *A*. *alba*. Also, splitting of two species (*S*. *alba* and *S*. *caseolaris*) formed additional OTUs (Fig 4A and 4B). The multiple threshold methods (mGMYC) gave two threshold time for ITS2 (-0.013035 and -0.005441) resulting into 9 clusters (CI:4–9) and 17 ML entities (CI:4–17). *matK*+ITS2 gave threshold time of -0.010859 and -0.004847, resulting into 9 clusters (CI:5–11) and 13 ML entities (CI:5–16). However, multiple thresholds overestimated the number of species, so we took a more conservative approach to consider only the results obtained from the single threshold (sGMYC) method. In GMYC, apart from other metrics, three unresolved species *R*. *apiculata*, *R*. *mucronata* and *A*. *alba* were distinctly resolved.

In addition to the above methods used for taxonomic evaluation, maximum likelihood (ML) based approach was added to get an additional perspective towards the species delineation through Poissons Tree Process (PTP). The ML analysis exhibited 10 OTUs with ITS2, where *Avicennia*, *Bruguiera*, *Rhizophora*, *Ceriops*, and *Kandelia* genera were merged while *S*. *alba* and *S*. *caseolaris* were split (Fig 4A and 4B). With *matK*+ITS2, 11 OTUs were formed by merging of *Avicennia*, *Bruguiera* and *Rhizophora* genera and *S*. *alba* was split.

## Discussion

There is no consensus regarding perfect plant DNA barcode, however few of plastid and nuclear coding (*rbcL*, *matK*, *rpoB*, and *rpoC1*) and non-coding (*trnH-psbA*, ITS2, *psbK-psbI* and *atpF-atpH*) marker fulfilled the required criteria [3, 9, 37]. The *rbcL* and *matK* are considered as core barcode, which can be further complemented with *trnH-psbA* and ITS2 as plant barcode suggested by China Plant BOL [4, 7]. We employed these markers for molecular identification of mangrove plant species. In our earlier report, we have tested potential barcode candidates *rbcL* and *matK* individual as well as concatenated *rbcL*+*matK*, which demarcated most of the species such as *A*. *ilicifolius*, *E*. *agallocha*, *A*. *corniculatum*, *K*. *candel*, *C*. *tagal*, *B*. *cylindrica* and *B*. *gymnorrhiza*. An initial analysis was performed based on traditional barcode methods (Barcode gap analysis and NJ tree with the K2P method) [17]. Individual, as well as concatenated *rbcL* and *matK* barcode demarcated almost all mangroves species except for *Rhizophora*, *Sonneratia* and *Avicennia* genera [17]. The Plant CBOL group (2009) reported that only 72% species were resolved using combined *rbcL* and *matK*. A similar result was observed after combining *rbcL* and *matK* from closely related species of *Curcuma* [13]. Moreover, *Avicennia* genera with three species, of which *A*. *alba*, was resolved perfectly using *matK* but *A*. *officinalis* and *A*. *marina* lumped together and unable to resolve at the species level. Low resolution using DNA barcode regions has been documented in many other plants such as the genus *Araucaria* (32%), *Solidago* (17%) and *Quercus* (0%) [38].

A high percentage of bidirectional reads were critical for a successful plant barcoding system, given the low amount of variation that separates many plant species [3–4]. The risk of misassignment can be anticipated due to sequencing error or incomplete bidirectional reads. We observed the significant quality of PCR amplification and sequencing ranged from 95% to 100% in all tested markers. However, for ITS2 barcode, we performed many amplifications and sequencing attempt for *S*. *alba*, *S*. *caseolaris*, and *A*. *ilicifolius* mangroves taxa. Sequencing of *S*. *alba* and *S*. *caseolaris* resulted in a mixed and low-quality chromatogram with unidirectional success. The possible explanation for this kind of situation can be underscored by the presence of either ITS as multiple copies or pseudogene or/and fungal ITS contamination in plant [39]. Species identification success rate using *rbcL+matK* is higher, whereas *rbcL* sequence recovery ranged from 90–100% [4, 38, 40]. Hence, CBOL group recommends *rbcL* primers to possess universality for land plants. As reported by CBOL, the *matK* region showed sequencing success of 90% [4]. The *matK* marker provided 88% sequencing success, with the use of 10 primer pair combinations [3].

Very few reports are available on the DNA barcoding of the mangrove taxa [17, 41]. Lower genetic distances were observed based on K2P among mangrove taxa particularly genera *Rhizophora*, *Sonneratia*, *Avicennia*, and *Bruguiera* based on *rbcL*, *matK* and ITS barcode [41]. Genetic distance ranged from 0.01 to 0.25 for *rbcL* gene, 0.01 to 0.89 for *matK* and 0.01–0.508 for ITS locus [41]. Similar results were observed in our studies, for *rbcL* and *matK* the genetic distance ranged from 0–0.68% and 0–1.32% respectively [17]. The discrimination power of proposed DNA barcode by CBOL Plant Working Group may vary in different plant group [12, 42–43]. Depending on the taxon, the use of additional markers may be needed for discrimination [4].

For single barcode ITS2, ABGD (K2P, X = 1), Taxon DNA (T = 3%) and GMYC produced consistent OTUs with corresponding results. Additionally, GMYC resolved *R*. *apiculata*, *R*. *mucronata*, and *A*. *alba* species. Overall highest taxon assignment was observed as 57.14% in GMYC and taxon resolution was up to 42.85% in ABGD, TaxonDNA, and PTP barcoding methods. However, the resolution of Chlorella-like species (microalgae) produced by GMYC, PTP, ABGD and character-based barcoding methods were variables based on several marker studies such as *rbcL*, ITS, and *tufA* [27]. Single ITS2 with PTP analysis was not able to resolve *C*. *tagal* and *K*. *candel*, which was further improved in the *matK*+ITS2 analysis. Analysis following the above methods, species delimitation through PTP and GMYC was utilized, due to their robustness in the absence of barcoding gap [44]. Even though they are based on different algorithms, both methods calculated the point of transition between species and population [27]. The GMYC method has a theoretically strong background and requires ultrametric gene tree that takes more time to analyse data. In contrast, the PTP is a recently developed method as an alternative to GMYC which requires non- ultrametric gene tree and consumes less time [44–45]. Both the methods revealed sort of identical results, however, the two analyses differed in resolution. In both the methods, five species (*B*. *cylindrica*, *B*. *gymnorrhiza*, *A*. *officinalis* and *A*. *marina*) in GMYC and seven species (*B*. *cylindrica*, *B*. *gymnorrhiza*, *A*. *alba*, *A*. *officinalis*, *A*. *marina*, *R*. *apiculata* and *R*. *mucronata*) in PTP were merged into single OTUs, potentially indicating low intraspecific diversity. It reflected that there are many overlooked/cryptic species present within the mangroves. When we performed ABGD with relative gap width X = 1.5 for K2P method, *S*. *alba*, and *S*. *caseolaris* species were demarcated, while rest of the mangrove species were split. At a relative gap width (X = 1) about seven species of the mangrove s were merged into single OTU and observed that the ABGD tends to lump species by increasing the number of merged OTUs [29]. Beside this, we also observed inconsistency of OTUs count during initial partition to recursive partition. Recursive partitioning recognizes more OTUs than initial ones, showing their superior capability to deal with variation in sample sizes of the species under study [29]. Moreover, TaxonDNA with a lower threshold value (0.3%) demarcated *B*. *cylindrica* and *B*. *gymnorrhiza*. The possible explanation for this might be due to lack of barcode gap resulting in merged OTUs, which can be optimized by analyzing more than 5 sequences per species, and we have used 3 sequences per species [28]. In TaxonDNA analysis, for *rbcL* threshold (T) was observed 0%, a similar result was recorded for *rbcL* in the Zingiberaceae family [46]. However, the threshold (T) for Indian Zingiberaceae family members was recorded as 0.20% for *rbcL* and 0% for *rpoB* and *accD* [43].

*Avicennia* is the most diverse mangrove genus, comprising eight species, out of which three are endemic to the Atlantic-East Pacific (AEP) region and five are endemic in the Indo-West Pacific region (IWP) [47]. A recent systematic revision of *Avicennia* based on morphological characters formed three groups: (1) *A*. *marina*; (2) *A*. *officinalis* and *A*. *integra*; and (3) *A*. *rumphiana* and *A*. *alba* [47]. In the current study, we have included *A*. *marina*, *A*. *officinalis*, and *A*. *alba* species, which were resolved with other barcode markers. Two plastid spacers such as *psbK-psbI* and *atpF-atpH* are recommended as potential plant DNA barcodes based on the flora of the Kruger National Park South Africa as a model system [48]. Similarly, we used three markers (*atpF-atpH*, *psbK-psbI* and *rpoC1*) for cryptic genera *Avicennia* and further evaluated with ABGD and TaxonDNA barcode methods. Both the methods consistently resolved all three *Avicennia* species using an *atpF-atpH* marker. Similarly, phylogenetic reconstruction of *Avicennia* genera based on *trnT-trnD* intergenic spacer region and the *psbA* gene revealed that *A*. *marina* is sister to the *A*. *officinalis*/*A*. *integra* and *A*. *alba* is genetically distinct [47].

## Conclusions

In the present study, we tested core DNA barcode *rbcL*, *ma*tK, ITS2, *atpF-atpH*, *psbK-psbI* and *rpoC1* to resolve mangroves species. Individual, as well as concatenated *matK*+ITS2 are helpful to demarcate mangroves at the species level. Single barcode *matK* is sufficient to resolve *A*. *ilicifolius*, *A*. *corniculatum*, *E*. *agallocha*, *Ceriops tagal*, *K*. *candel*, *B*. *cylindrica and B*. *gymnorrhiza*. ITS2 was able to discriminate *R*. *apiculata* and *R*. *mucronata* species based on GMYC method, while *A*. *alba* was resolved by concatenation of *matK*+ITS2. A cryptic genus *Avicennia* was delimitated based on the *atpF-atpH* single barcode. In the present work, the foundation work was done towards DNA barcoding of mangroves plant genera, such as *Rhizophora*, *Avicennia*, *Acanthus*, *Kandelia*, *Ceriops*, *Bruguiera*, *Aegiceras* and *Excoecaria*. Compiled mangroves barcoding result had some limitations, most of which are due to the low mangrove taxa sample coverage. Further, there is a need to explore additional mangroves taxa which will improve mangrove species identification for practical conservation.

## Supporting information

S1 Table List of primers used in the current study. (DOCX)

S2 Table Automated Barcode Gap Discovery web server based analysis of all barcodes (*matK*, ITS2, *matK*+ITS2, *atpF-atpH*, *psbK-psbI* and *rpoC1* using two relative gap width (X = 1 and 1.5) and three different matrices such as JC, K2P, and p-simple distance. (DOCX)

S1 Fig Neighbor-joining tree (Kimura 2 Parameter distance using bootstrap value of 1000 replicates) *matK+*ITS2 concatenated NJ (K2P). (DOCX)

## References

[1] Hebert PDN, Ratnasingham S, deWaard JR. Barcoding animal life: cytochrome c oxidase subunit 1 divergences among closely related species. Proc R Soc Biol Sci SerB. 2003; 270: S96–S99. 10.1098/rsbl.2003.0025 PMC1698023 12952648

[2] Hebert PDN, Cywinska A, Ball SL, deWaard JR. Biological identifications through DNA barcodes. Proc R Soc Biol Sci SerB. 2003; 270: 313–321. 10.1098/rspb.2002.2218 PMC1691236 12614582

[3] Fazekas AJ, Burgess KS, Kesanakurti PR, Graham SW, Newmaster SG, Husband BC, Percy DM, Hajibabaei M, Barrett SC. Multiple multilocus DNA barcodes from the plastid genome discriminate plant species equally well. PLoS One. 2008; 3: e2802 doi: 10.1371/journal.pone.0002802 18665273 10.1371/journal.pone.0002802 PMC2475660

[4] CBOL Plant Working Group. A DNA barcode for land plants. Proc Natl Acad Sci USA. 2009; 106: 12794–12797. doi: 10.1073/pnas.0905845106 19666622 10.1073/pnas.0905845106 PMC2722355

[5] Kress WJ, Wurdack KJ, Zimmer EA, Weigt LA, Janzen DH. Use of DNA barcodes to identify flowering plants. Proc Natl Acad Sci USA. 2005; 102: 8369–8374. doi: 10.1073/pnas.0503123102 15928076 10.1073/pnas.0503123102 PMC1142120

[6] Hollingsworth PM, Graham SW, Little DP. Choosing and using a plant DNA barcode. PLoS One. 2011; 6: e19254 doi: 10.1371/journal.pone.0019254 21637336 10.1371/journal.pone.0019254 PMC3102656

[7] China Plant BOL Group, Li DZ, Gao LM, Li HT, Wang H, Ge XJ, et al Comparative analysis of a large dataset indicates that internal transcribed spacer (ITS) should be incorporated into the core barcode for seed plants. Proc Natl Acad Sci USA. 2011; 108: 19641–19646. doi: 10.1073/pnas.1104551108 22100737 10.1073/pnas.1104551108 PMC3241788

[8] Chen S, Yao H, Han J, Liu C, Song J, et al Validation of the ITS2 region as a novel DNA barcode for identifying medicinal plant species. PLoS One. 2010; 5: e8613 doi: 10.1371/journal.pone.0008613 20062805 10.1371/journal.pone.0008613 PMC2799520

[9] Kress J, Erickson DL. A two-locus global DNA barcode for land plants: The coding *rbcL* gene complements the non-coding *trnH-psbA* spacer region. PLoS One. 2007; 2: e508 doi: 10.1371/journal.pone.0000508 17551588 10.1371/journal.pone.0000508 PMC1876818

[10] Newmaster SG, Fazekas AJ, Steeves RAD, Janovec J. Testing candidate plant barcode regions in the Myristicaceae. Mol Ecol Resour. 2008; 8: 480–490. doi: 10.1111/j.1471-8286.2007.02002.x 21585825 10.1111/j.1471-8286.2007.02002.x

[11] Burgess KS, Fazekas AJ, Kesanakurti PR, Graham SW, Husband BC, et al Discriminating plants species in a local temperate flora using the *rbcL*+*matK* DNA barcode. Method Ecol Evol. 2011; 2: 333–340.

[12] Li HQ, Chen JY, Wang S, Xiong SZ. Evaluation of six candidate DNA barcoding loci in Ficus (Moraceae) of China. Mol Ecol Resour. 2012; 12: 783–790. doi: 10.1111/j.1755-0998.2012.03147.x 22537273 10.1111/j.1755-0998.2012.03147.x

[13] Chen J, Zhao J, Erickson DL, Xia N, Kress WJ. Testing DNA barcodes in closely related species of *Curcuma* (Zingiberaceae) from Myanmar and China. Mol Ecol Resour. 2015; 15: 337–348. doi: 10.1111/1755-0998.12319 25158042 10.1111/1755-0998.12319

[14] Xiang XG, Hu H, Wang W, Jin XH. DNA barcoding of the recently evolved genus *Holcoglossum* (Orchidaceae: Aeridinae): a test of DNA barcode candidates. Mol Ecol Resour. 2011; 11: 1012–1021. doi: 10.1111/j.1755-0998.2011.03044.x 21722327 10.1111/j.1755-0998.2011.03044.x

[15] Zhang CY, Wang FY, Yan HF, Hao G, Hu CM, et al Testing DNA barcoding in closely related groups of *Lysimachia* L. (Myrsinaceae). Mol Ecol Resour. 2012; 12: 98–108. doi: 10.1111/j.1755-0998.2011.03076.x 21967641 10.1111/j.1755-0998.2011.03076.x

[16] Tomlinson PB. The botany of mangroves. 2nd ed. Cambridge; Cambridge University Press; 1986.

[17] Saddhe AA, Jamdade RA, Kumar K. Assessment of mangroves from Goa, west coast India using DNA barcode. SpringerPlus. 2016; 5: 1554 doi: 10.1186/s40064-016-3191-4 27652127 10.1186/s40064-016-3191-4 PMC5021661

[18] Shi S, Huang Y, Zeng K, Tan F, He H, et al Molecular phylogenetic analysis of mangroves: independent evolutionary origins of vivipary and salt secretion. Mol Phylogenet Evol. 2006; 40: 298–304. 15579389 10.1016/j.ympev.2004.09.002

[19] Spalding M, Kainuma M, Collins L. World atlas of mangroves. Earthscan eBook; 2010.

[20] Polidoro BA, Carpenter KE, Collins L, Duke NC, Ellison AM, et al The loss of species: mangrove extinction risk and geographic areas of global concern. PLoS One. 2010; 5: e10095 doi: 10.1371/journal.pone.0010095 20386710 10.1371/journal.pone.0010095 PMC2851656

[21] Kathiresan K, Bingham BL. Biology of mangroves and mangrove ecosystems. Advances in Marine Biology. 2001; 40: 25–81.

[22] Naskar K, Mandal R. Ecology and Biodiversity of Indian Mangroves. Daya Publishing House New Delhi; 1999.

[23] Tamura K, Stecher G, Peterson D, Filipski A.Kumar S. MEGA6: Molecular Evolutionary Genetics Analysis version 6.0. Mol Biol Evo. 2013; l30: 2725–2729. 10.1093/molbev/mst197 PMC3840312 24132122

[24] Ratnasingham S, Hebert PDN. BOLD: The Barcode of Life Data System (www.barcodingoflife.org). Mol Ecol Notes. 2007; 7: 355–364. doi: 10.1111/j.1471-8286.2007.01678.x 18784790 10.1111/j.1471-8286.2007.01678.x PMC1890991

[25] Rozas J. Polymorphism Analysis using DnaSP In Posada D, editors. Bioinformatics for DNA Sequence Analysis: Methods in Molecular Biology Series, Humana Press NJ USA 2009 pp. 337–350. 10.1007/978-1-59745-251-9_17 19378153

[26] Meier R, Kwong S, Vaidya G, Ng PKL. DNA barcoding and taxonomy in diptera: a tale of high intraspecific variability and low identification success. Syst Biol. 2006; 55: 715–728. doi: 10.1080/10635150600969864 17060194 10.1080/10635150600969864

[27] Zou S, Fei C, Song J, Bao Y, He M, Wang C. Combining and comparing coalescent, distance and character-based approaches for barcoding microalgaes: A Test with Chlorella-like species (Chlorophyta). PLoS One. 2016; 11: e0153833 doi: 10.1371/journal.pone.0153833 27092945 10.1371/journal.pone.0153833 PMC4841637

[28] Puillandre N, Lambert A, Brouillet S, Achaz G. ABGD, Automatic Barcode Gap Discovery for primary species delimitation. Mol ecol. 2012; 21: 1864–77. doi: 10.1111/j.1365-294X.2011.05239.x 21883587 10.1111/j.1365-294X.2011.05239.x

[29] Yang Z, Landry J-F, Hebert PDN. A DNA Barcode Library for North American Pyraustinae (Lepidoptera: Pyraloidea: Crambidae). PLoS One. 2016; 11: e0161449 doi: 10.1371/journal.pone.0161449 27736878 10.1371/journal.pone.0161449 PMC5063472

[30] Drummond AJ, Rambaut A. BEAST: Bayesian evolutionary analysis by sampling trees. BMC Evol Biol. 2007; 7: 214 doi: 10.1186/1471-2148-7-214 17996036 10.1186/1471-2148-7-214 PMC2247476

[31] Drummond AJ, Ho SY, Phillips MJ, Rambaut A. Relaxed phylogenetics and dating with confidence. PLoS Biol. 2006; 4: e88 doi: 10.1371/journal.pbio.0040088 16683862 10.1371/journal.pbio.0040088 PMC1395354

[32] Gernhard T. The conditioned reconstructed process. J Theoret Biol. 2008; 253: 769–778. 18538793 10.1016/j.jtbi.2008.04.005

[33] Kumar S, Skjaeveland A, Orr RJS, Enger P, Ruden T, et al AIR: a batch-oriented web program package for construction of supermatrices ready for phylogenomic analyses. BMC Bioinfo. 2009; 10: 357. 10.1186/1471-2105-10-357 PMC2777179 19863793

[34] R Core Team. R: A Language and Environment for Statistical Computing. R Foundation for Statistical Computing, Vienna, Austria. 2012.

[35] Paradis E, Claude J, Strimmer K. APE: analyses of phylogenetics and evolution in R language. Bioinfo. 2004; 20: 289–290. 10.1093/bioinformatics/btg412 14734327

[36] Ezard T, Fujisawa T, Barraclough TG. splits: SPecies’ LImits by Threshold Statistics. 2009. R package version. 1.0-14/r31. (http://R-Forge.R-project.org/projects/splits/).

[37] Pennisi E. Taxonomy. Wanted: A barcode for plants. Science. 2007; 318: 190–191. doi: 10.1126/science.318.5848.190 17932267 10.1126/science.318.5848.190

[38] Little DP, Stevenson DW. A comparison of algorithms for the identification of specimens using DNA barcodes: examples from gymnosperms. Cladistics. 2007; 23: 1–21. 10.1111/j.1096-0031.2006.00126.x 34905841

[39] Álvarez I, Wendel JF. Ribosomal ITS sequences and plant phylogenetic inference. Mol phyl evol. 2003; 29: 417–34. 10.1016/s1055-7903(03)00208-2 14615184

[40] Ross HA, Murugan S, Li WLS. Testing the reliability of genetic methods of species identification via simulation. Syst Biol. 2008; 57: 216–230. doi: 10.1080/10635150802032990 18398767 10.1080/10635150802032990

[41] Sahu SK, Singh R, Kathiresan K. Multi-gene phylogenetic analysis reveals the multiple origin and evolution of mangrove physiological traits through exaptation. Estuarine, Coastal and Shelf Science. 2016; 183: 41–51.

[42] Hollingsworth ML, ANDRA CLARK AL, Forrest LL, Richardson J, Pennington R, et al Selecting barcoding loci for plants: Evaluation of seven candidate loci with species-level sampling in three divergent groups of land plants. Mol Ecol Res. 2009; 9: 439–457. 10.1111/j.1755-0998.2008.02439.x 21564673

[43] Vinitha RM, Kumar SU, Aishwarya K, Sabu M, Thomas G. Prospects for discriminating Zingiberaceae species in India using DNA barcodes. J. Integr Plant Biol. 2014; 56: 760–773. doi: 10.1111/jipb.12189 24612741 10.1111/jipb.12189

[44] Tang CQ, Humphreys AM, Fontaneto D, Barraclough TG. Effects of phylogenetic reconstruction method on the robustness of species delimitation using single‐locus data. Methods in Ecol Evol. 2014; 5: 1086–1094. 10.1111/2041-210X.12246 PMC4374709 25821577

[45] Dumas P, Barbut J, Le Ru B, Silvain JF, Clamens AL, et al Phylogenetic molecular species delimitations unravel potential new species in the pest genus Spodoptera Guenée, 1852 (Lepidoptera, Noctuidae). PLoS one. 2015; 10: e0122407 doi: 10.1371/journal.pone.0122407 25853412 10.1371/journal.pone.0122407 PMC4390195

[46] Chen J, Zhao J, Erickson DL, Xia N, Kress WJ. Testing DNA barcodes in closely related species of *Curcuma* (Zingiberaceae) from Myanmar and China. Mol Ecol Resour. 2015; 15: 337–348. doi: 10.1111/1755-0998.12319 25158042 10.1111/1755-0998.12319

[47] Li X, Duke NC, Yang Y, Huang L, Zhu Y, et al Re-Evaluation of phylogenetic relationships among species of the mangrove genus *Avicennia* from Indo-West Pacific based on multilocus analyses. PLoS One. 2016; 11: e0164453 doi: 10.1371/journal.pone.0164453 27716800 10.1371/journal.pone.0164453 PMC5055292

[48] Lahaye R, Savolainen V, Duthoit S, Maurin O, van der Bank M. A test of *psbK-psbI* and *atpF-atpH* as potential plant DNA barcodes using the flora of the Kruger National park as a model system (South Africa). Nature Precedings. 2008; 1–21.

