# Supplementary material for: Evaluation of multilocus marker efficacy for delineating mangrove species of West Coast India
Source: PLoS One. 2017 Aug 17;12(8):e0183245. doi: 10.1371/journal.pone.0183245 (PMC5560660; doi:10.1371/journal.pone.0183245)
Supplement: S1 Table — (DOCX) [file pone.0183245.s001.docx]

**S1 Table.** List of primers used in the current study.

| **Marker** | **Sequence** |
| --- | --- |
| ITS2-S2F | ATGCGATACTTGGTGTGAAT |
| ITS4 | TCCTCCGCTTATTGATATGC |
| *atpF* | ACTCGCACACACTCCCTTTCC |
| *atpH* | GCTTTTATGGAAGCTTTAACAAT |
| *psbK* | TTAGCCTTTGTTTGGCAAG |
| *psbI* | AGAGTTTGAGAGTAAGCAT |
| *rpoC1 2f* | GGCAAAGAGGGAAGATTTCG |
| *rpoC1 4r* | CCATAAGCATATCTTGAGTTGG |
