# Supplementary material for: Evaluation of multilocus marker efficacy for delineating mangrove species of West Coast India
Source: PLoS One. 2017 Aug 17;12(8):e0183245. doi: 10.1371/journal.pone.0183245 (PMC5560660; doi:10.1371/journal.pone.0183245)
Supplement: S2 Table — (DOCX) [file pone.0183245.s002.docx]

**S2 Table.** Automated Barcode Gap Discovery web server based analysis of all barcodes (*matK*, ITS2, *matK*+ITS2, *atpF-atpH, psbK-psbI* and *rpoC1* using two relative gap width (X=1 and 1.5) and three different matrices such as JC, K2P and p-simple distance.

|  | Relative gap width | Prior intraspecific distance | Jukes-Cantor (JC69) | | K2P | | p-Distance | |
| --- | --- | --- | --- | --- | --- | --- | --- | --- |
|  |  |  | Initial partition | Recursive partition | Initial partition | Recursive partition | Initial partition | Recursive partition |
| ITS2 | X=1.0 | 0.1 | 2 | 2 | 2 | 2 | 2 | 2 |
|  |  | 0.059948 | 7 | 7 | 7 | 7 | 7 | 7 |
|  |  | 0.035938 | 13 | 13 | 7 | 7 | 7 | 7 |
|  |  | 0.021544 | 13 | 13 | 12 | 12 | 13 | 13 |
|  |  | 0.012915 | 13 | 13 | 12 | 12 | 13 | 13 |
|  |  | 0.007743 | 13 | 13 | 12 | 12 | 13 | 13 |
|  |  | 0.004642 | 13 | 14 | 12 | 13 | 13 | 14 |
|  |  | 0.002783 | 13 | 14 | 12 | 13 | 13 | 14 |
|  |  | 0.001668 | 13 | 19 | 12 | 18 | 13 | 14 |
|  |  | 0.001 | 13 | 19 | 12 | 18 | 13 | 14 |
|  | X=1.5 | 0.1 | 2 | 2 | 2 | 2 | 2 | 2 |
|  |  | 0.059948 | 7 | 7 | 7 | 7 | 7 | 7 |
|  |  | 0.035938 | 7 | 10 | 7 | 7 | 7 | 7 |
|  |  | 0.021544 | 7 | 10 | 7 | 10 | 7 | 10 |
|  |  | 0.012915 | 7 | 10 | 7 | 10 | 7 | 10 |
|  |  | 0.007743 | 7 | 10 | 7 | 10 | 7 | 10 |
|  |  | 0.004642 | 7 | 11 | 7 | 11 | 7 | 11 |
|  |  | 0.002783 | 7 | 11 | 7 | 11 | 7 | 11 |
|  |  | 0.001668 | 7 | 16 | 7 | 16 | 7 | 11 |
|  |  | 0.001 | 7 | 16 | 7 | 16 | 7 | 11 |
| *matK* | X=1.0 | 0.1 | 4 | 5 | 4 | 5 | 4 | 5 |
|  |  | 0.059948 | 4 | 5 | 4 | 5 | 4 | 5 |
|  |  | 0.035938 | 6 | 6 | 6 | 6 | 6 | 6 |
|  |  | 0.021544 | 6 | 6 | 6 | 6 | 6 | 6 |
|  |  | 0.012915 | 8 | 9 | 8 | 9 | 8 | 8 |
|  |  | 0.007743 | 8 | 10 | 8 | 10 | 8 | 9 |
|  |  | 0.004642 | 8 | 11 | 8 | 10 | 8 | 10 |
|  |  | 0.002783 | 8 | 11 | 8 | 11 | 8 | 10 |
|  |  | 0.001668 | 8 | 11 | 8 | 11 | 8 | 10 |
|  |  | 0.001 | 8 | 15 | 8 | 15 | 8 | 10 |
|  | X=1.5 | 0.1 | 0 | 1 | 0 | 0 | 0 | 1 |
|  |  | 0.059948 | 4 | 5 | 0 | 1 | 4 | 5 |
|  |  | 0.035938 | 6 | 6 | 6 | 6 | 6 | 6 |
|  |  | 0.021544 | 6 | 6 | 6 | 6 | 6 | 6 |
|  |  | 0.012915 | 6 | 7 | 6 | 7 | 6 | 6 |
|  |  | 0.007743 | 6 | 10 | 6 | 10 | 6 | 9 |
|  |  | 0.004642 | 6 | 11 | 6 | 10 | 6 | 10 |
|  |  | 0.002783 | 6 | 11 | 6 | 11 | 6 | 10 |
|  |  | 0.001668 | 6 | 11 | 6 | 11 | 6 | 10 |
|  |  | 0.001 | 6 | 25 | 6 | 16 | 6 | 12 |

| **ITS2*+***  ***matK*** | X=1.0 | 0.1 |  | 1 |  | 1 |  | 1 |
| --- | --- | --- | --- | --- | --- | --- | --- | --- |
|  |  | 0.059948 | 4 | 5 | 4 | 5 | 4 | 5 |
|  |  | 0.035938 | 6 |  | 6 |  | 6 |  |
|  |  | 0.021544 | 6 |  | 6 |  | 6 |  |
|  |  | 0.012915 | 11 |  | 11 |  | 11 |  |
|  |  | 0.007743 | 11 |  | 11 |  | 11 |  |
|  |  | 0.004642 | 11 | 12 | 11 |  | 11 |  |
|  |  | 0.002783 | 11 | 12 | 11 |  | 11 |  |
|  |  | 0.001668 | 11 | 13 | 11 |  | 11 |  |
|  |  | 0.001 | 11 | 13 | 11 |  | 11 |  |
|  | X=1.5 | 0.1 |  | 1 |  | 1 |  | 1 |
|  |  | 0.059948 | 4 | 5 | 4 | 5 | 4 | 5 |
|  |  | 0.035938 | 6 |  | 6 |  | 6 |  |
|  |  | 0.021544 | 6 |  | 6 |  | 6 |  |
|  |  | 0.012915 | 6 | 8 | 6 | 8 | 6 | 8 |
|  |  | 0.007743 | 6 | 9 | 6 | 9 | 6 | 8 |
|  |  | 0.004642 | 6 | 10 | 6 | 10 | 6 | 9 |
|  |  | 0.002783 | 6 | 10 | 6 | 10 | 6 | 10 |
|  |  | 0.001668 | 6 | 11 | 6 | 10 | 6 | 11 |
|  |  | 0.001 | 6 | 11 | 6 | 11 | 6 | 11 |

| ***atpF-atpH*** | X=1.0 | 0.0046 | 1 |  | 1 |  | NA |  |
| --- | --- | --- | --- | --- | --- | --- | --- | --- |
|  |  | 0.002783 | 3 |  | 3 |  |  |  |
|  |  | 0.001668 | 3 |  | 3 |  |  |  |
|  |  | 0.001 | 3 |  | 3 |  |  |  |
|  | X=1.5 | 0.0046 |  | 1 |  | 1 | NA |  |
|  |  | 0.002783 | 3 |  | 3 |  |  |  |
|  |  | 0.001668 | 3 |  | 3 |  |  |  |
|  |  | 0.001 | 3 |  | 3 |  |  |  |
| ***psbK-psbI*** | X=1.0 | 0.0129 | 0 | 1 |  |  | 0 | 1 |
|  |  | 0.0077 | 2 |  |  |  | 2 |  |
|  |  | 0.0046 | 2 |  | 1 |  | 2 |  |
|  |  | 0.002783 | 4 |  | 4 |  | 2 |  |
|  |  | 0.001668 | 4 |  | 4 |  | 2 |  |
|  |  | 0.001 | 4 |  | 4 |  | 2 |  |
|  | X=1.5 | 0.0129 | 0 | 1 | NA |  | 0 | 1 |
|  |  | 0.0077 | 2 |  |  |  | 2 |  |
|  |  | 0.0046 | 2 |  |  |  | 2 |  |
|  |  | 0.002783 | 4 |  |  |  | 2 |  |
|  |  | 0.001668 | 4 |  |  |  | 2 |  |
|  |  | 0.001 | 4 |  |  |  | 2 |  |
| ***rpoC1*** | X=1.0 | 0.002783 |  | 1 |  | 1 | NA |  |
|  |  | 0.001668 | 2 |  | 2 |  |  |  |
|  |  | 0.001 | 2 |  | 2 |  |  |  |
|  | X=1.5 | 0.002783 | NA |  | NA |  | NA |  |
|  |  | 0.001668 |  |  |  |  |  |  |
|  |  | 0.001 |  |  |  |  |  |  |
