# Supplementary material for: Evaluation of multilocus marker efficacy for delineating mangrove species of West Coast India
Source: PLoS One. 2017 Aug 17;12(8):e0183245. doi: 10.1371/journal.pone.0183245 (PMC5560660; doi:10.1371/journal.pone.0183245)

**S1 Fig.** Neighbor joining tree (Kimura 2 Parameter distance using bootstrap value of 1000 replicates) *matK+*ITS2 concatenated NJ (K2P).


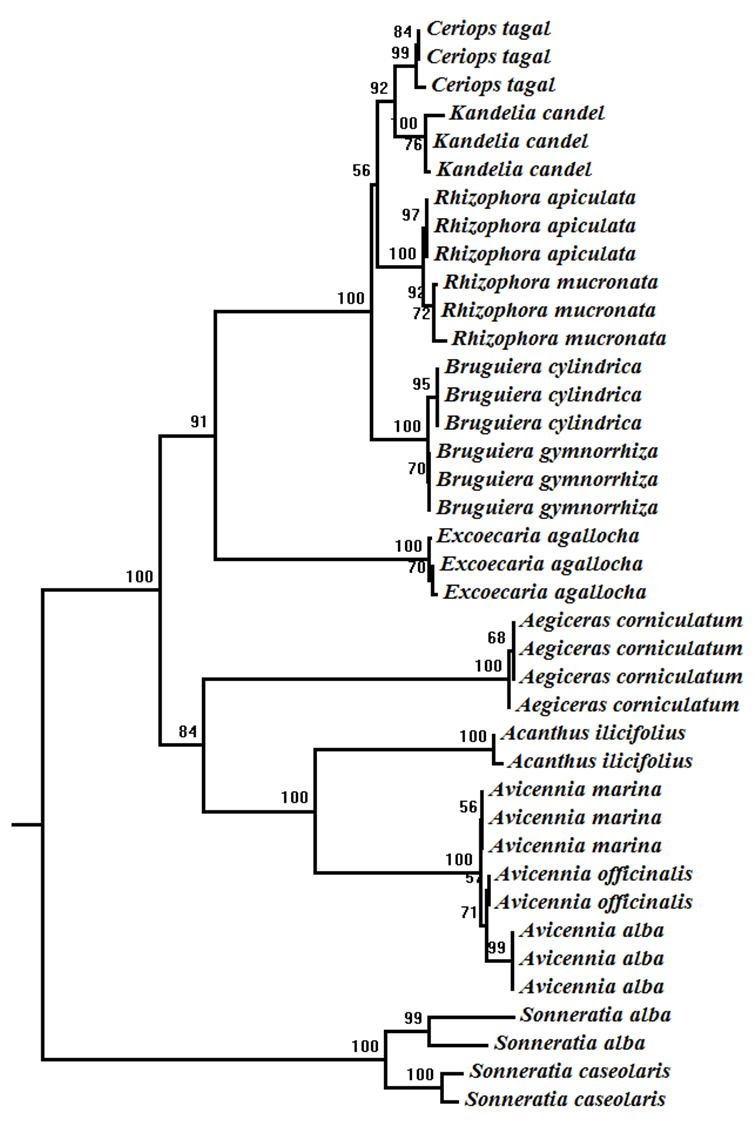

Supplement: S1 Fig — (DOCX) [file pone.0183245.s003.docx]
